# CC and CXC chemokine levels in children with meningococcal sepsis accurately predict mortality and disease severity

**DOI:** 10.1186/cc4836

**Published:** 2006-02-20

**Authors:** Clementien L Vermont, Jan A Hazelzet, Ester D de Kleijn, Germie PJM van den Dobbelsteen, Ronald de Groot

**Affiliations:** 1 Department of Pediatrics, Erasmus MC-Sophia Children's Hospital, Rotterdam, The Netherlands; 2 Netherlands Vaccine Institute, Laboratory for Vaccine Development, Bilthoven, The Netherlands

## Abstract

**Introduction:**

Chemokines are a superfamily of small peptides involved in leukocyte chemotaxis and in the induction of cytokines in a wide range of infectious diseases. Little is known about their role in meningococcal sepsis in children and their relationship with disease severity and outcome.

**Methods:**

Monocyte chemoattractant protein (MCP)-1, macrophage inflammatory protein (MIP) 1α, growth-related gene product (GRO)-α and interleukin (IL)-8 were measured in 58 children with meningococcal sepsis or septic shock on admission and 24 hours thereafter. Nine patients died. Serum chemokine levels of survivors and nonsurvivors were compared, and the chemokine levels were correlated with prognostic disease severity scores and various laboratory parameters.

**Results:**

Extremely high levels of all chemokines were measured in the children's acute-phase sera. These levels were significantly higher in nonsurvivors compared with survivors and in patients with septic shock compared with patients with sepsis (*P *< 0.0001). The cutoff values of 65,407 pg/ml, 85,427 pg/ml and 460 pg/ml for monocyte chemoattractant protein, for IL-8 and for macrophage inflammatory protein 1α, respectively, all had 100% sensitivity and 94–98% specificity for nonsurvival. Chemokine levels correlated better with disease outcome and severity than tumor necrosis factor (TNF)-α and correlated similarly to interleukin (IL)-6. In available samples 24 hours after admission, a dramatic decrease of chemokine levels was seen.

**Conclusion:**

Initial-phase serum levels of chemokines in patients with meningococcal sepsis can predict mortality and can correlate strongly with disease severity. Chemokines may play a key role in the pathophysiology of meningococcal disease and are potentially new targets for therapeutic approaches.

## Introduction

*Neisseria meningitidis *is one of the most feared causative agents in childhood infectious diseases, mainly affecting children below the age of four and adolescents. It can cause meningitis, sepsis and septic shock, characterized by a rapid development of petechiae or purpura fulminans. Meningococcal lipopolysaccharide, a constituent of the bacterial outer membrane, plays a central role in the pathophysiology of meningococcal sepsis. The release of large amounts of lipopolysaccharide into the blood stream induces a cascade of reactions by the host immune response, including massive activation of the complement system, activation of the coagulation system and the induction of proinflammatory and anti-inflammatory cytokines. High levels of these inflammatory mediators, such as tumor necrosis factor alpha (TNF-α) and IL-6, are associated with disease fatality.

Chemokines belong to a family of more than 40 relatively small peptides, which are involved in chemoattraction and activation of leukocytes to the site of inflammation and in the induction of cytokine production. Chemokines are thus key determinants of inflammatory reactions and immunity [1-3]. These peptides are secreted by tissue cells, leucocytes and activated epithelial cells [4]. Four different subfamilies can be identified based on the highly conserved presence of the first two cysteine residues, which are either separated or not by other amino acids: the CC chemokines, the CXC chemokines, the CX_3_C chemokines and the C chemokines [5]. Chemokines act through a family of chemokine receptors, which are present on cell types such as leukocytes, dendritic cells and endothelial cells.

CXC chemokines, which include growth-related gene product alpha (GRO-α) and IL-8, are potent chemoattractants for neutrophils, whereas the CC chemokines, including monocyte chemoattractant protein 1 (MCP-1) and macrophage inflammatory protein 1α (MIP-1α), attract monocytes, lymphocytes, basophils, eosinophils and natural killer cells. The C and CX_3_C chemokine families are represented by only one chemokine each: lymphotactin and fractalkine, respectively. Lymphotactin is thought to be mainly involved in chemoattraction of lympocytes whereas fractalkine, a membrane-bound molecule expressed on endothelial cells, mediates the capturing and adhesion of circulating leucocytes [6,7].

Chemokines and their receptors play an important role in the innate immunity against infectious diseases such as HIV/AIDS and malaria, but also play an important role in autoimmune diseases [8,9]. The role of chemokines in meningococcal sepsis or septic shock has not so far been studied intensively. In meningococcal disease, lipo-oligosaccharide and outer membrane proteins of the meningococcus induce a strong inflammatory response in patients. Studies in patients with bacterial meningitis, caused by *N. meningitidis*, *Streptococcus pneumoniae *or *Haemophilus influenzae*, showed high levels of IL-8 and MCP-1 in cerebrospinal fluid and variably increased levels of GRO-α and MIP-1α [10-13]. In studies of chemokines in patients with meningococcal sepsis or septic shock, only serum levels of IL-8 and RANTES (regulated on activation, normal T cell expressed and secreted) have been reported. IL-8 levels are positively correlated with disease severity and outcome, as opposed to RANTES, which is significantly lower in patients with severe disease and in nonsurvivors [14-16].

The aim of this study was to measure the serum levels of CXC and CC chemokines during the initial phase of meningococcal sepsis in children and to determine their relationship with disease severity and outcome.

## Materials and methods

### Patients

Children with a clinical diagnosis of meningococcal sepsis or septic shock were included between July 1997 and March 2000 and between December 2001 and July 2002 after written informed consent was obtained from their parents or legal guardians. This retrospective study was approved by the medical ethics committee of Erasmus MC.

Inclusion criteria for meningococcal sepsis were: age between 1 month and 18 years, a petechial rash and/or purpura fulminans, tachycardia, tachypnea and a body temperature <36°C or >38.5°C. Inclusion criteria for meningococcal septic shock were all of the aforementioned and either persistent hypotension despite adequate volume supplementation or two or more features of poor end-organ perfusion: pH ≤ 7.3, base deficit <-5 or plasma lactate >2.0 mmol/l; arterial hypoxia defined as pO_2 _<75 mmHg, a pO_2_/FiO_2 _ratio <250 or TcSaO_2 _saturation <96% in patients without pre-existing pulmonary disease, acute renal failure defined as urine output <0.5 ml/kg/hour for at least 1 hour despite adequate fluid volume loading and without renal disease, or a sudden deterioration of baseline mental status not resulting from meningitis [17].

As soon as possible, but at least within six hours after admission to the pediatric intensive care unit, blood was drawn from an arterial line and serum and plasma samples were collected and stored at -80°C until assays were performed. For this study, either serum or plasma was used to measure chemokines by means of ELISA. When the arterial line was still present, blood was again drawn 24 hours after inclusion and serum or plasma samples were collected and stored.

Thirty-eight children with a meningococcal septic shock participated in a randomized, placebo-controlled dose-finding study of protein C concentrate [18]. In this study children received either placebo or one of three dosages of protein C concentrate every 6 hours for the first 3 days, followed by every 12 hours with a maximum of 7 days. Serum samples used in the present study were drawn just before infusion of the study medication and 24 hours after the start of the treatment.

### Assays

Serum levels of GRO-α, MIP-1α and MCP-1 were measured by ELISA (Quantikine; R&D Systems (Minneapolis, MN, USA) according to the manufacturer's instructions. Samples were first diluted 1:2 in the appropriate buffer and, when chemokine concentrations of chemokines were above the upper limit of the standard curve of the assay, additional dilutions up to 1:500 were made. The lower detection limit for GRO-α, MIP-1α and MCP-1 was 20 pg/ml. IL-8 levels were also measured by ELISA (Sanquin, Amsterdam, The Netherlands). Clinical data were collected at inclusion, and the Pediatric Risk of Mortality (PRISM) score, the Sepsis-related Organ Failure Assessment score (adapted for pediatric use) and the Disseminated Intravascular Coagulation score were assessed for all patients on admittance to determine the disease severity [19-21]. Laboratory parameters including white blood cell counts, lactate concentrations and serum C-reactive protein were measured on admission.

### Statistical analysis

Clinical scores and parameters of patients are presented as means and 95% confidence intervals (CIs). For statistical analysis, samples with chemokine levels below the detection limit were assessed as the value of the detection limit of the assays. Chemokine levels are presented as the median, percentiles and ranges. Differences in chemokine levels between survivors and nonsurvivors were analyzed by Mann-Whitney *U* tests. Differences in chemokine levels between different time points were analyzed by the Wilcoxon Signed Ranks test. Receiver-operating characteristic curves were calculated for all chemokines to determine the optimum cutoff values in predicting disease outcome. Correlations between chemokine levels and disease severity parameters were investigated by calculating Spearman's rho correlation coefficient (*r*_s_). All tests were two-tailed and *P *< 0.05 was considered significant.

## Results

### Patients

Fifty-eight patients were included, of which six had a meningococcal sepsis and 52 had septic shock according to the criteria. The median age of the patients was 4.0 years (range 0.1–16.1 years). Nine patients died of septic shock (15.5%), all but one within 24 hours of admission. Thirty-seven patients needed ventilatory support at the time of first sampling (64%). The mean PRISM score on admission was 22.0 (95% CI, 19.6–24.4), the mean Sepsis-related Organ Failure Assessment score was 10.2 (95% CI, 9.0–11.3) and the mean Disseminated Intravascular Coagulation score was 5.1 (95% CI, 4.6–5.7). The mean lactate concentration was 4.4 mmol/l (95% CI, 3.8–5.0), the mean C-reactive protein level was 96 mg/l (95% CI, 77–114) and the mean white blood cell count was 11.6 × 10^9^/l (95% CI, 9.4–13.8). The mean interval between the onset of symptoms and the time of first blood sampling was 13.9 hours (95% CI, 11.5–16.4).

### Chemokines

MCP-1 and IL-8 were detectable in all patient samples at inclusion with a median value of 5,340 pg/ml (range 91–445,600 pg/ml) and 9,541 pg/ml (range, 28–427,500 pg/ml), respectively. MIP-1α was detectable in 33 out of 58 patients (57%) with a median value of 164 pg/ml (range, 20–9,784 pg/ml), and GRO-α levels were detectable in 40 patients (69%) with a median value of 892 pg/ml (range, 20–101,150 pg/ml). MIP-1α, GRO-α and MCP-1 levels were significantly higher in patients with septic shock than those in patients with sepsis (*P *= 0.009, *P *= 0.005 and *P *= 0.006, respectively), whereas there was no significant difference in IL-8 levels between patients with sepsis and septic shock (*P *= 0.066).

**Figure 1:**
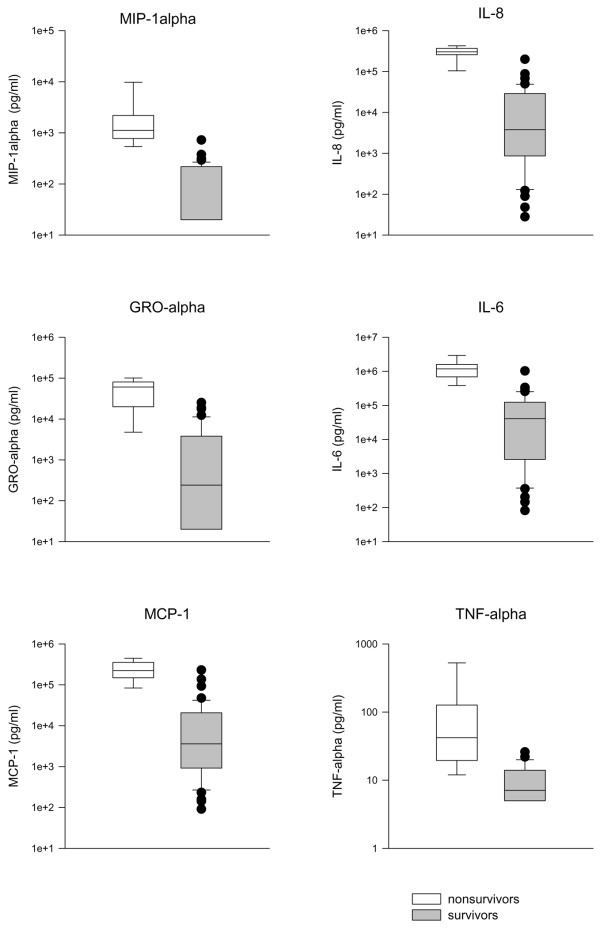
Levels of growth-related gene product alpha (GRO-α), monocyte chemoattractant protein 1 (MCP-1), macrophage inflammatory protein 1α (MIP-1α), IL-8, tumor necrosis factor alpha (TNF-α) and IL-6 in survivors versus nonsurvivors of meningococcal sepsis or septic shock. Black lines in boxes represent median values, boxes represent interquartile ranges, bars represent the 10th and 90th percentiles, and black dots are outlying values.

Chemokine levels on admission strongly correlated with each other, as well as with levels of IL-6 and TNF-α with Spearman correlation coefficients ranging from 0.56 to 0.91 (data not shown). Significant differences were seen between survivors and nonsurvivors (Mann-Whitney *U* test, *P *< 0.0001) for all serum chemokine levels, as well as for the cytokines TNF-α and IL-6 (Figure 1). All nonsurvivors had higher levels of MCP-1, MIP-1α, IL-8 and IL-6 compared with survivors. Using receiver-operating characteristic curve analysis, cutoff values of 65,407 pg/ml for MCP-1, 460 pg/ml for MIP-1α, 85,427 pg/ml for IL-8 and 361 ng/ml for IL-6 were determined, which all had 100% sensitivity and a specificity between 94% and 98% in predicting nonsurvival.

**Figure 2:**
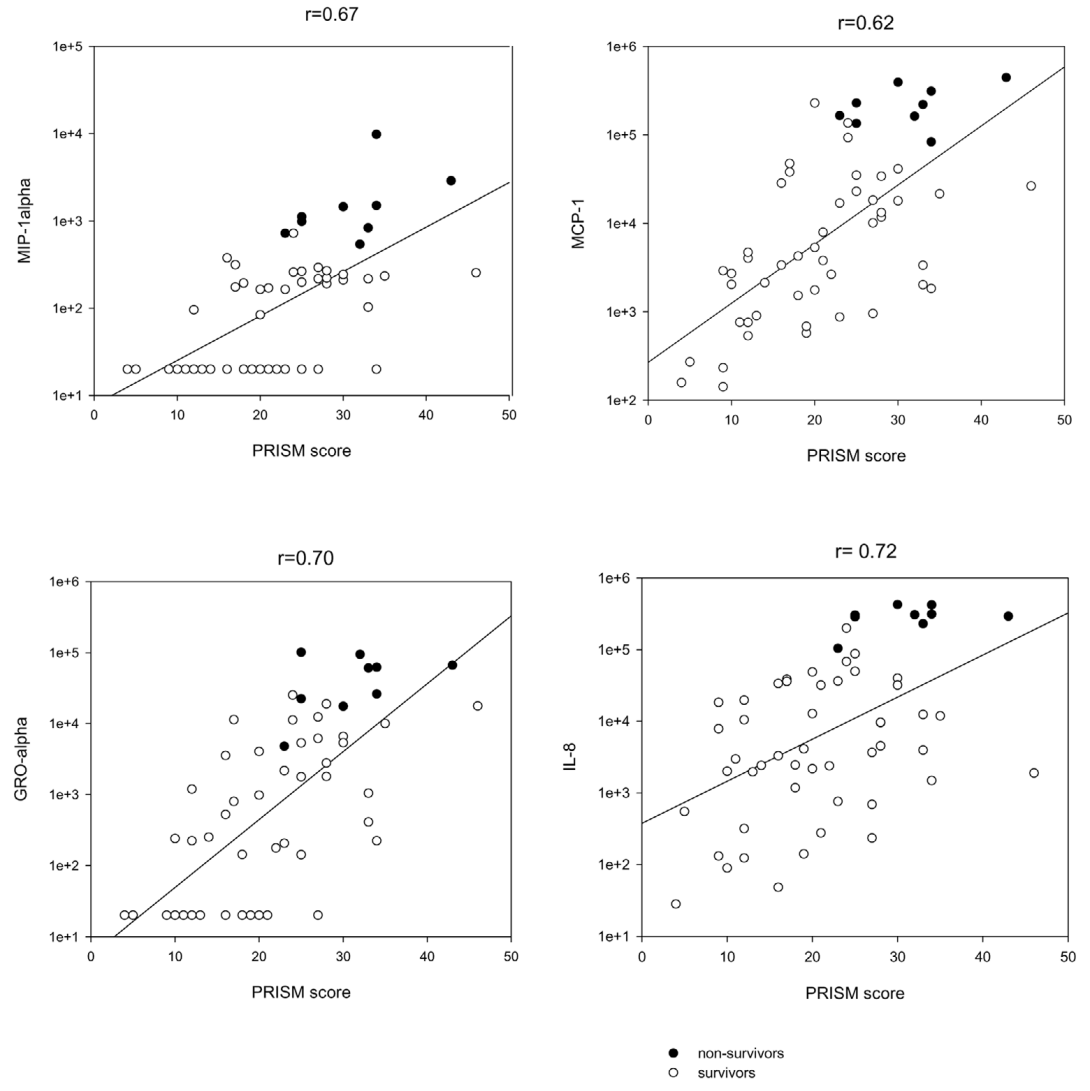
Correlation between chemokine levels in serum samples of children with meningococcal sepsis or septic shock and Pediatric Risk of Mortality (PRISM) scores on admission. The horizontal lines in the charts for macrophage inflammatory protein 1α (MIP-1α) and growth-related gene product alpha (GRO-α) indicate the detection limit for the assay. MCP-1, monocyte chemoattractant protein 1.

We found positive correlations between chemokine levels and PRISM scores (IL-8, *r*_s _= 0.72; MIP-1α, *r*_s _= 0.67; GRO-α, *r*_s _= 0.70; and MCP-1, *r*_s _= 0.62; *P *< 0.0001) (Figure 2). Correlation coefficients between MIP-1α, GRO-α and MCP-1 and PRISM scores were higher than between TNFα levels and PRISM scores (*r*_s _= 0.56) and were slightly lower than between IL-6 and PRISM scores (*r*_s _= 0.73). High correlations were also found between serum chemokine levels and Disseminated Intravascular Coagulation scores, Sepsis-related Organ Failure Assessment scores and laboratory parameters for disease severity and activation of coagulation such as lactate concentration, C-reactive protein and white blood cell counts, D-dimers and fibrinogen levels (Table 1). Furthermore, initial serum levels of MCP-1 and MIP-1α, but not of GRO-α and IL-8, were negatively correlated with the interval between the appearance of petechiae and the time of blood sampling (both *r*_s _= -0.28, *P *= 0.037).

**Figure 3:**
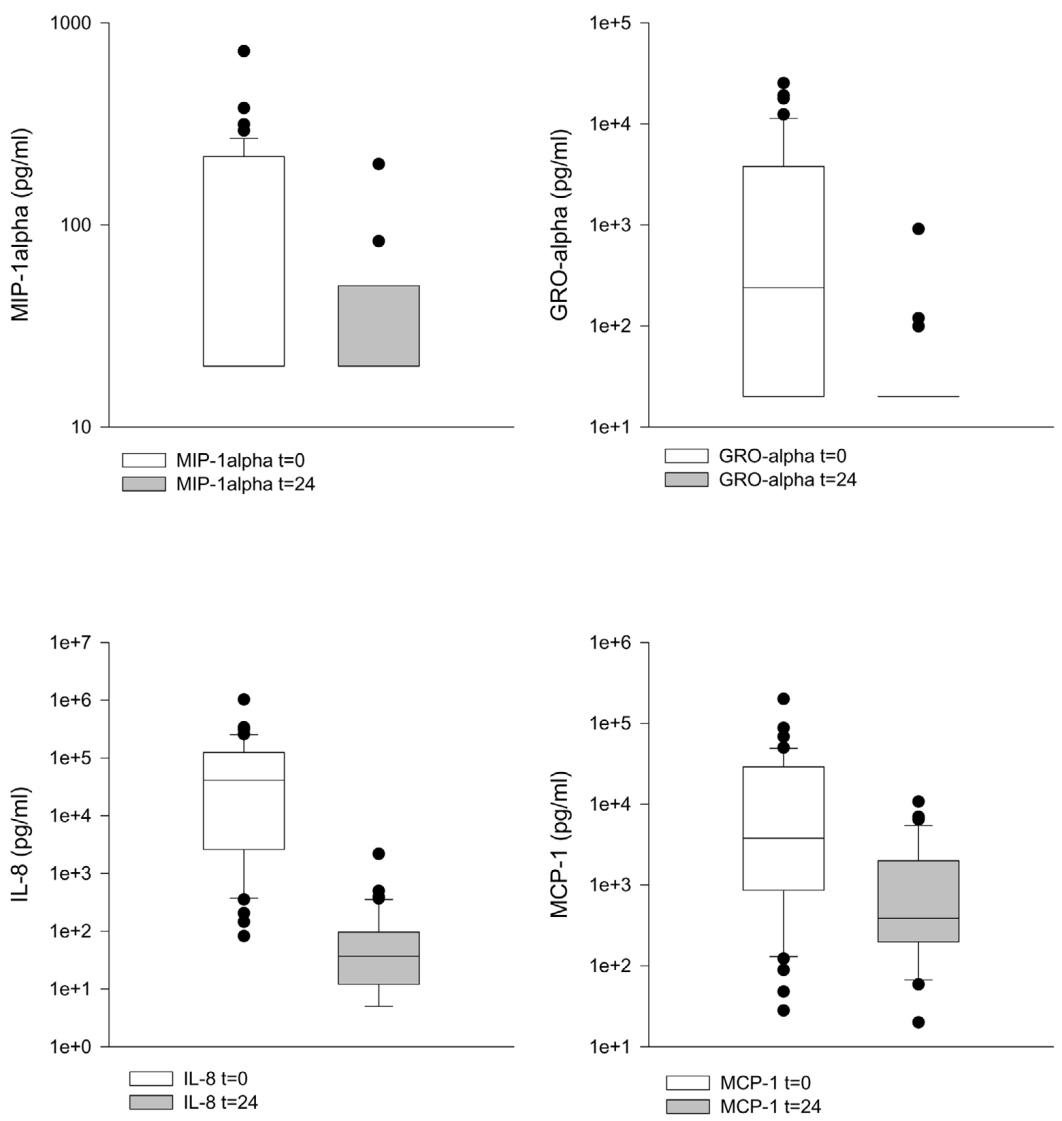
Chemokine levels in serum samples of children with meningococcal sepsis or septic shock on admission and levels obtained 24 hours after admission to the pediatric intensive care unit (pg/ml). Black lines in boxes represent median values, boxes represent interquartile ranges, bars represent the 10th and 90th percentiles, and black dots are outlying values. GRO-α, growth-related gene product alpha; MCP-1, monocyte chemoattractant protein 1; MIP-1α, macrophage inflammatory protein 1α.

Eight out of nine nonsurvivors died within 24 hours after admission. Chemokine levels in available sera of children 24 hours after pediatric intensive care unit admission (*n *= 49) showed a significant decrease (*P *< 0.0001 for all chemokines) (Figure 3).

## Discussion

A complex network of cytokines, complement factors and coagulation and fibrinolysis factors are involved in the pathophysiology of meningococcal sepsis as a response to the very high loads of lipopolysaccharide and meningococcal outer membrane proteins. Chemokines are involved in directing leucocytes to the site of inflammation and are probably necessary for the translation of the innate immune response against pathogens into a specific acquired response [22]. The present study demonstrates the presence of extremely high levels of chemokines from the CC family as well as the CXC family in sera obtained from children in the initial phase of meningococcal sepsis or septic shock. This implies a generalized upregulation of both chemokine families in the early stage of meningococcal disease in children. Schinkel and colleagues showed earlier that chemokines of both families are readily produced within 2 hours after endotoxin challenge in healthy volunteers [23].

Median levels of IL-8, MCP-1 and MIP-1α were higher than those described in patients with meningococcal meningitis [10,11]. Furthermore, peak MIP-1α levels were 20 times higher and IL-8 levels were 250 times higher than levels described in adult patients with sepsis [24].

MIP-1α and GRO-α were moderately elevated or even undetectable in moderately ill patients, but reached very high levels in the severely ill patients. MCP-1 and IL-8 serum levels were detectable in all patients and reached very high levels in severely ill patients, especially in nonsurvivors. These results are in accordance with those described by Møller and colleagues, who showed that levels of MCP-1, IL-8 and MIP-1α were significantly higher in patients with fulminant meningococal septicemia as compared with patients with distinct meningitis or mild disease [25]. In addition, we show strong significant differences between survivors and nonsurvivors in serum levels of MCP-1, MIP-1α and IL-8. Cutoff values with 100% sensitivity and 94–98% specificity in predicting outcome were calculated for these chemokines. This is in contrast to TNF-α, probably the most intensively studied cytokine in meningococcal disease, for which a cutoff value of 22.5 had a 78% sensitivity and 98% specificity. IL-6, another cytokine known to be involved in meningococcal sepsis, was also higher in all nonsurvivors than in survivors [26,27]. In our study, IL-6 had a cutoff value with a similar sensitivity and specificity as chemokines.

The inverse correlation between MCP-1 and MIP-1α and the interval between the appearance of petechiae suggests that these chemokines play a major role in severely ill patients in whom the course of disease is more rapid than in other patients. Chemokine levels also correlated with disease severity, as indicated by the high correlations between disease severity scores and laboratory parameters. Correlations between chemokine levels and PRISM scores were higher than correlations between TNF-α and PRISM scores, indicating that serum levels of these chemokines are a better predictor for disease severity than TNF-α. Common polymorphisms in the MIP-1α, MCP-1 and IL-8 genes have been recently discovered and are associated with an increased production of these chemokines [28-30]. Further research is needed to determine the role of these genetic polymorphisms in the severity of meningococcal disease in children.

The results of our study suggest that chemokine levels may be suitable candidates for implementation in prognostic scores based on laboratory parameters. Chemokines may play a key role in the pathophysiology of meningococcal disease, and chemokines as well as their receptors are potentially new targets for therapeutic approaches. Chemokine receptor antagonists, antichemokine antibodies and broad-spectrum chemokine inhibitors are currently under development [31,32]. Future research will have to show their applicability in meningococcal disease.

## Conclusion

Serum levels of CXC and CC chemokines in children in the initial phase of meningococcal sepsis can predict disease severity and outcome.

## Key messages

• 	Extremely high levels of CC chemokines as well as CXC chemokines are found in sera from children in the initial phase of meningococcal sepsis.

• 	Chemokine levels in children in the initial phase of meningococcal sepsis accurately predict disease severity and outcome.

## Abbreviations

CI = confidence interval; ELISA = enzyme-linked immunosorbent assay; GRO-α = growth-related gene product alpha; IL = interleukin; MCP-1 = monocyte chemoattractant protein 1; MIP-1α = macrophage inflammatory protein 1α; PRISM = Pediatric Risk of Mortality; RANTES = regulated on activation, normal T cell expressed and secreted; TNF-α = tumor necrosis factor alpha.

## Competing interests

The authors declare that they have no competing interests.

## Authors' contributions

CLV collected patient samples and data, carried out the ELISA experiments, performed the statistical analysis of the study and drafted the manuscript. JAH participated in the design and coordination of the study and helped write the manuscript. EDdK participated in the collection of patient samples and data. GPJMvdD participated in the design of the study, coordinated and supervised the laboratory experiments and helped write the manuscript. RdG conceived of the study and participated in its design.

## Figures and Tables

**Table 1:** Spearman correlation coefficients between serum chemokine levels, laboratory parameters for disease severity and disease severity scores on admission

	Sepsis-related Organ Failure Assessment score	Disseminated Intravascular Coagulation score	Lactate	White blood cells	C-reactive protein	D-dimers	Fibrinogen
IL-8	0.62	0.70	0.41	-0.57	-0.35	0.53	-0.69
MIP-1α	0.79	0.72	0.59	-0.66	-0.47	0.64	-0.83
GRO-α	0.78	0.72	0.52	-0.70	-0.43	0.66	-0.81
MCP-1	0.68	0.78	0.51	-0.70	-0.50	0.60	-0.79

GRO-α, growth-related gene product alpha; MCP-1, monocyte chemoattractant protein 1; MIP-1α, macrophage inflammatory protein 1α.
